# Krebs von den Lungen-6 glycoprotein circulating levels are not useful as prognostic marker in COVID-19 pneumonia: A large prospective cohort study

**DOI:** 10.3389/fmed.2022.973918

**Published:** 2022-08-08

**Authors:** Ivan Castellví, Diego Castillo, Hèctor Corominas, Anaís Mariscal, Sandra Orozco, Natividad Benito, Virginia Pomar, Andrés Baucells, Isabel Mur, David de la Rosa-Carrillo, David Lobo, Ana Milena Millan, Nerea Hernández de Sosa, David Filella, Laia Matas, Laura Martínez-Martínez, Cándido Juarez, Jordi Casademont, Pere Domingo

**Affiliations:** ^1^Department of Rheumatology and Systemic Autoimmune Diseases, Hospital de la Santa Creu i Sant Pau, Barcelona, Spain; ^2^Department of Pneumology, Hospital Universitari de la Santa Creu i Sant Pau, Barcelona, Spain; ^3^Department of Immunology, Hospital Universitari de la Santa Creu i Sant Pau, Barcelona, Spain; ^4^Division of Infectious Diseases, Hospital Universitari de la Santa Creu i Sant Pau, Barcelona, Spain; ^5^Department of Internal Medicine, Hospital de la Santa Creu i Sant Pau, Barcelona, Spain

**Keywords:** COVID-19, Krebs von den Lungen-6 (KL-6), pneumonia, biomarker, predictor

## Abstract

Coronavirus disease 2019 (COVID-19) has rapidly expanded worldwide. Currently, there are no biomarkers to predict respiratory worsening in patients with mild to moderate COVID-19 pneumonia. Small studies explored the use of Krebs von de Lungen-6 circulating serum levels (sKL-6) as a prognostic biomarker of the worsening of COVID-19 pneumonia. We aimed at a large study to determine the prognostic value of sKL-6 in predicting evolving trends in COVID-19. We prospectively analyzed the characteristics of 836 patients with COVID-19 with mild lung disease on admission. sKL-6 was obtained in all patients at least at baseline and compared among patients with or without respiratory worsening. The receiver operating characteristic curve was used to find the optimal cutoff level. A total of 159 (19%) patients developed respiratory worsening during hospitalization. Baseline sKL-6 levels were not higher in patients who had respiratory worsening (median {IQR} 315.5 {209–469} vs. 306 {214–423} U/ml *p* = 0.38). The last sKL-6 and the change between baseline and last sKL-6 were higher in the respiratory worsening group (*p* = 0.02 and *p* < 0.0001, respectively). The best sKL-6 cutoff point for respiratory worsening was 497 U/ml (area under the curve 0.52; 23% sensitivity and 85% specificity). sKL-6 was not found to be an independent predictor of respiratory worsening. A conditional inference tree (CTREE) was not useful to discriminate patients at risk of worsening. We found that sKL-6 had a low sensibility to predict respiratory worsening in patients with mild-moderate COVID-19 pneumonia and may not be of use to assess the risk of present respiratory worsening in inpatients with COVID-19 pneumonia.

## Introduction

Since severe acute respiratory syndrome coronavirus 2 (SARS-CoV-2) was identified as the cause of coronavirus disease 2019 (COVID-19) in Wuhan, China at the end of December 2019 (1), it has expanded rapidly, causing a global pandemic with more than 400 million confirmed cases and a death toll of more than five million people, according to the World Health Organization (1) (https://covid19.who.int).

The clinical manifestations of COVID-19 are variable. Most affected patients show mild respiratory symptoms without special healthcare requirements (2, 3). Nevertheless, some patients require hospital admission due to respiratory failure caused by severe pneumonia (4). Several therapies, including corticoids, Janus kinase inhibitors, interleukin-6 receptor antagonists, and antivirals (remdesivir, molnupiravir, and nirmatrelvir), improve the course of the disease (5), but only vaccines have demonstrated the strength required to modify this scenario (6). Despite this, SARS-CoV-2 infection can still lead to adult respiratory distress syndrome (ARDS), especially in immunocompromised patients (7). Different poor prognosis factors have been identified, such as age, male sex, chronic comorbidities, and laboratory parameters (8–14).

The mucin-like glycoprotein Krebs von den Lungen-6 (KL-6) is primarily expressed on the surface of type II epithelial alveolar cells when the cell is damaged or regenerating (13). KL-6 has been proposed as a potentially useful biomarker in Interstitial Lung Diseases (ILDs) (15–20). sKL-6 has also been described as a biomarker for pulmonary damage in ARDS since higher levels correlate well with mean and peak airway pressure (21). The usefulness of the sKL-6 level as a prognostic biomarker in COVID-19 has been suggested in previous cohort studies but their interpretation is limited due to the sample size (from 5 to 364 cases) (22–28).

Our study aimed to assess the potential of sKL-6 as a biomarker of lung disease progression in a large cohort of COVID-19 cases and to determine its prognostic value.

## Methods

### Study population

A total of 876 patients, admitted consecutively to a tertiary university hospital in the COVID-19 emergency context, were included in a prospective, observational, single-center study. All patients had a SARS-CoV-2 infection, laboratory-confirmed by a real-time reverse transcription-polymerase chain reaction (PCR) test using nasopharyngeal or oropharyngeal swabs, and had mild to moderate COVID-19 pneumonia without baseline needs for intensive care unit (ICU) admittance, high oxygen flux supplementation, or noninvasive ventilation. The clinical status of our admitted patients was assessed using the WHO eight-category ordinal scale (29): (1) not hospitalized and no limitations of activities; (2) not hospitalized, with limitation of activities, home oxygen requirement, or both; (3) hospitalized, not requiring supplemental oxygen, and no longer requiring ongoing medical care (used if hospitalization was extended for infection-control or other nonmedical reasons); (4) hospitalized and not requiring supplemental oxygen but requiring ongoing medical care (related to COVID-19 or to other medical conditions); (5) hospitalized and requiring any supplemental oxygen; (6) hospitalized, requiring noninvasive ventilation, or use of high-flow oxygen supply; (7) hospitalized, receiving invasive mechanical ventilation, or extracorporeal membrane oxygenation (ECMO); and (8) death. Patients with a clinical status corresponding to categories 4 or 5 at admission were included in this study.

All patients were admitted between 14 March 2020 and 7 February 2021. Most patients (55.3%) were recruited during the first and second COVID-19 waves and received hydroxychloroquine 400 mg/12 h (day 1), hydroxychloroquine 200 mg/12 h (days 2–5), and azithromycin 500 mg/day (days 1–3), according to our standard of care at that time point. In patients with cytokine release syndrome (CRS), defined as respiratory function worsening and high or progressively increasing D-dimer (>1,500 ng/ml) or high IL-6 levels (>40 pg/ml), a single dose of intravenous tocilizumab (600 mg for patients ≥75 kg; 400 mg for those <75 kg) was prescribed following Spanish Agency for Medicinal Products and Medical Devices (AEMPS) recommendations (https://www.aemps.gob.es/la-aemps/ultima-informacion-de-la-aemps-acerca-del-covid%e2%80%9119/tratamientos-disponibles-para-el-manejo-de-la-infeccion-respiratoria-por-SARS-CoV-2/?lang=en). Furthermore, all patients received a prophylactic low dose of molecular weight heparin unless otherwise contraindicated. Oral consent was obtained from patients due to the pandemic emergency situation, in line with our observational study design. The ethics committee of the Hospital Universitari de la Santa Creu i Sant Pau approved this study (IIBSP-COV-2020-35).

### Data collection

At least one serum sample for KL-6 was obtained from each patient during hospital admission. In patients with long-standing admission, more KL-6 samples were collected based on the criteria of the attending physician. Respiratory function worsening was considered if patients changed to a worse category of the WHO eight-category ordinal scale during hospitalization. The PaO_2_/FiO_2_ (PAFI) ratio was estimated according to the method developed by Brown et al. (30, 31).

Demographic data, preexisting chronic medical conditions (including presence or absence of lung diseases and/or connective tissue diseases), previous use of potential toxic lung drugs, clinical symptoms at hospitalization, length of symptoms, length of hospital stay, and clinical and laboratory (complete blood count, renal and liver function, C-reactive protein, D-dimer, and ferritin) outcomes during hospitalization, including the time to worsening since KL-6 serum extraction, of the need for invasive mechanical ventilation, ICU admission, length of hospital stay, and in-hospital death, were collected from each patient. We analyzed all patients with at least one sKL-6 and performed an additional analysis in patients with serial sKL-6 levels.

### KL-6 assay

sKL-6 levels were determined in serum samples using the chemiluminescence reagent “Lumipulse G KL-6” (Fujirebio Europe NV, Gent, Belgium) following the manufacturer's instructions and expressed in U/ml.

### Statistical analysis

Continuous or interval data are summarized in terms of the number of observations, mean and standard deviation (SD), median, minimum, maximum, and interquartile range (IQR). Differences in continuous variables between groups (worsening vs. not worsening) were performed using a Student's *t*-test or Mann-Whitney *U* test (nonparametric) when the data did not meet the assumptions of normality as evaluated using the Kolmogorov-Smirnov test. Nominal and ordinal categorical data were summarized in terms of the number of subjects providing data at the relevant time point (*n*), frequency counts, and percentages presented to one decimal place. Differences between groups for nominal data were identified using the Fisher-exact test. The *p*-value between groups for ordinal data was estimated using the exact Mantel-Haenszel test. The sensitivity, specificity, positive predictive value (PPV), negative predictive value (NPV), accuracy, and error rate of all KL-6 values for respiratory worsening were calculated by the area under the receiver operating characteristic (ROC) curve (AUC). The optimal cutoff KL-6 value was obtained using the Youden's index. Independent predictive factors of respiratory worsening were obtained using the multivariate logistic regression model. The final model selects predictive factors as those independent variables with a *p* < 0.05 on the Wald test. The odds ratio (OR) and the 95% confidence interval (95% CI) were calculated with the exponential model coefficients. The predictive accuracy of the logistic regression model was evaluated using the classification table, where observed values for the dependent outcome and the predicted values (at a defined cutoff value, e.g., *p* = 0.50) were cross-classified, showing for each predicted value the % of correct events, % correct nonevents, sensitivity, specificity, PPV, and NPV. Time to worsening from admission and from the first symptom was defined as the time from admission (or first symptom) until the date of worsening. Patients without worsening were censored at the hospital discharge date. The median days of time free of worsening, with its 95%CI, were estimated using the Kaplan-Meier (KM) survival analysis and using KL-6 optimal cutoff point, and the statistical significance between groups was performed using the log-rank test. Prognostic factors of time (days) to worsening were obtained using the multivariate cox regression model, with all potential prognostic factors, including KL-6 optimal cutoff, as independent variables. Thereafter, independent variables reaching a *p* < 0.05 were included in the final adjusted model. The adjusted median days of time free of worsening, with its 95%CI, were estimated using the KL-6 optimal cutoff point. The *p*-value and the 95% confidence interval for the hazard ratio (HR) were estimated based on the Wald test. Additionally, unbiased conditional inference trees (CTREE) were fitted to analyze the associations between worsening and all covariates at baseline (Initial visit 0), including demographic data, previous pathologies, clinical signs, and analytic data. CTREE is a conditional recursive partitioning algorithm that solves both the overfitting problem and the variable selection bias present in other recursive partitioning algorithms (32). All the variables are potential candidates to be included in the model. The variable selection process is automated, and no assumptions regarding the underlying structure and distribution are needed. As a result, the tree shows the variables in a hierarchical structure of the model that has a relative real weight for taking decisions.

A *p* < 0.05 was considered statistically significant.

All report outputs were produced using SAS^®^ version 9.4 (TS1M5) in a secure and validated environment. Copyright (c) 2016 by SAS Institute Inc., Cary, NC, USA.

## Results

Of 876 patients, 836 patients (487 men) met the inclusion criteria. Their median age was 56 (IQR, 46–64) years, and 273 (66.1%) were older than 50 years of age. Of 836 patients, 461 patients (55.1%) had at least one comorbidity, with hypertension (31.9%) and dyslipidemia (24.9%) being the most frequent. Also, 267 patients (31.4%) had a history of tobacco use, and 152 (15.8%) had previous lung diseases. The use of drugs with potential lung toxicity (chemotherapy, immunosuppressants, and angiotensin-converting enzyme inhibitors) was documented in 17 (2%) patients. The most frequent presenting symptom was fever (*n* = 645, 77.2%), followed by cough (*n* = 544, 65.1%). All patients had ill-defined consolidations on chest X-rays, but only 400 (47.8%) reported dyspnea, while 50 (6.0%) had oxygen saturation by pulse oximeter (pSatO2) <93% on their first clinical exam. Patient baseline characteristics are summarized in Table 1.

**Table 1:** Patient characteristics at hospital admission.

**Respiratory worsening during hospitalization n (%)**	**Yes**	**No**	**Total**	***p***
	**159 (19)**	**677 (81)**	**836 (100)**	
Female *n* (%)	61 (38.4)	288 (42.5)	171 (45.6)	ns
Age (median [IQR])	59 [53–67]	56 [44–63]	56 [46–64]	0.0001
Comorbidities *n* (%)	100 (62.9)	361 (53.3)	461 (55.1)	0.034
Hypertension	60 (37.7)	207 (30.6)	267 (31.9)	0.09
Dyslipidemia	47 (29.6)	178 (26.3)	225 (26.9)	ns
Diabetes	20 (12.6)	99 (14.6)	119 (14.3)	ns
Lung disease	33 (20.8)	99 (14.6)	132 (15.8)	0.069
SAD	10 (6.3)	660 (97.5)	809 (96.8)	0.023
Lung Toxics Exposure *n* (%)				
Tobacco	43 (27)	168 (24.8)	211 (25.2)	ns
Drugs	8 (5)	9 (1.3)	17 (2)	0.0074
Symptoms *n* (%)				
Fever (≥37.3°C)	129 (81.1)	516 (76.2)	645 (77.2)	ns
Dyspnea	92 (57.9)	308 (45.5)	400 (47.8)	0.0061
Cough	96 (60.4)	448 (66.2)	544 (65.1)	ns
O2 Sat <93% n (%)	21 (13.2)	29 (4.3)	50 (6)	0.0001
% O2 Sat (median [IQR])	95 [94–96]	96 [95–97]	96 [95–97]	<0.0001
P/F Ratio (median [IQR])mmHg	304 [271–338]	329[295–375]	326 [290–367]	<0.0001
Laboratory parameters				
Leucocytes, 10^9^/L	7.4 ± 4.3	6.7 ± 3.6	6.8 ± 3.7	0.0408
Lymphocytes,10^9^/L	1.1 ± 0.7	1.2 ± 1.7	1.2 ± 1.4	0.0078
Platelets, 10^9^/L	199 ± 91.5	214 ± 90.3	211.2 ± 90.7	0.0068
CRP, mg/L	113.5 ± 79.5	80.8 ± 72.1	87 ± 74.6	<0.0001
LDH, U/L	357.8 ± 136.5	339.3 ± 662.4	342.8 ± 600.3	<0.0001
ALT, U/L	42.5 ± 29.1	43.8 ± 48.1	43.6 ± 45.1	ns
AST, U/L	43.2 ± 23.6	41.7 ± 42.5	42 ± 39.7	0.0014
GGT, U/L	90.1 ± 108.4	80.8 ± 91.1	82.8 ± 94.9	ns
D–dimer, mg/L	1,673 ± 6,661.6	1,335.7 ± 7,030	1,398.2 ± 6,959.7	ns
Ferritin, ug/L	1,174.5 ± 1,341.6	815.2 ± 908	882.1 ± 1,009.8	0.041
sKL−6 (median[IQR])	315.5 [209–469]	306[214–423]	307 [211–430]	ns

IQR, interquartile range; SAD, systemic autoimmune disease; O2 Sat, oxygen saturation; P/F ratio, arterial oxygen partial pressure to fractional inspired oxygen ratio; CRP, C- reactive protein; LDH, lactate dehydrogenase; ALT, alanine aminotransferase; AST, aspartate aminotransferase; GGT, gamma-glutamyltransferase; sKL-6, serum Krebs von den Lungen-6.

The median time between symptoms and hospitalization was 7 (IQR, 5-10) days, and the median hospital stay was 6 (IQR, 4-9) days. All patients were admitted between the first and third COVID-19 waves in Spain, and national treatment protocols changed during that period. In total, 405 (48.4%) and 422 (50.5%) patients received hydroxychloroquine and azithromycin, respectively. Also, 169 patients (20.4%) received tocilizumab, and corticosteroids were prescribed in 232 patients (27.8%).

A total of 159 patients (19%) developed respiratory function worsening during their hospital stay. Also, 17 patients (2.0%) died, and 13 patients were in the worsening respiratory group. The four deaths in the nonworsening respiratory group were related to previous cancer complications (50%) and aspirative pneumonia (50%).

### Serum KL-6 concentrations

A total of 1,952 samples of sKL-6 from 836 patients were collected. All patients provided at least one sKL-6 sample. Notably, 419, 288, 207, 153, and 49 patients had two, three, four, five, or six sKL-6 samples, respectively. Median baseline sKL-6 was 307 (IQR, 211-430; range was 15.8–6,295 U/ml), and the last sKL-6 values were 368 (IQR, 254-542; range 1.5–2,202 U/ml).

### Baseline sKL-6 circulating levels and respiratory function worsening

There was no difference in baseline sKL-6 levels in patients with or without respiratory function worsening (median {IQR} 315.5 {209–469} vs. 306 {214–423} U/ml, *p* = 0.39) and last sKL-6 values increased significantly in both groups (*p* < 0.0001). Nevertheless, delta (Δ) sKL-6 (median 95 {29–259} vs. 36 {5–113} U/ml, *p* < 0.0001) and the last sKL-6 levels were higher in patients that worsened (median {IQR} 414 {292–683.5} vs. 364 {250–530} U/ml, *p* = 0.027).

**Figure 1:**
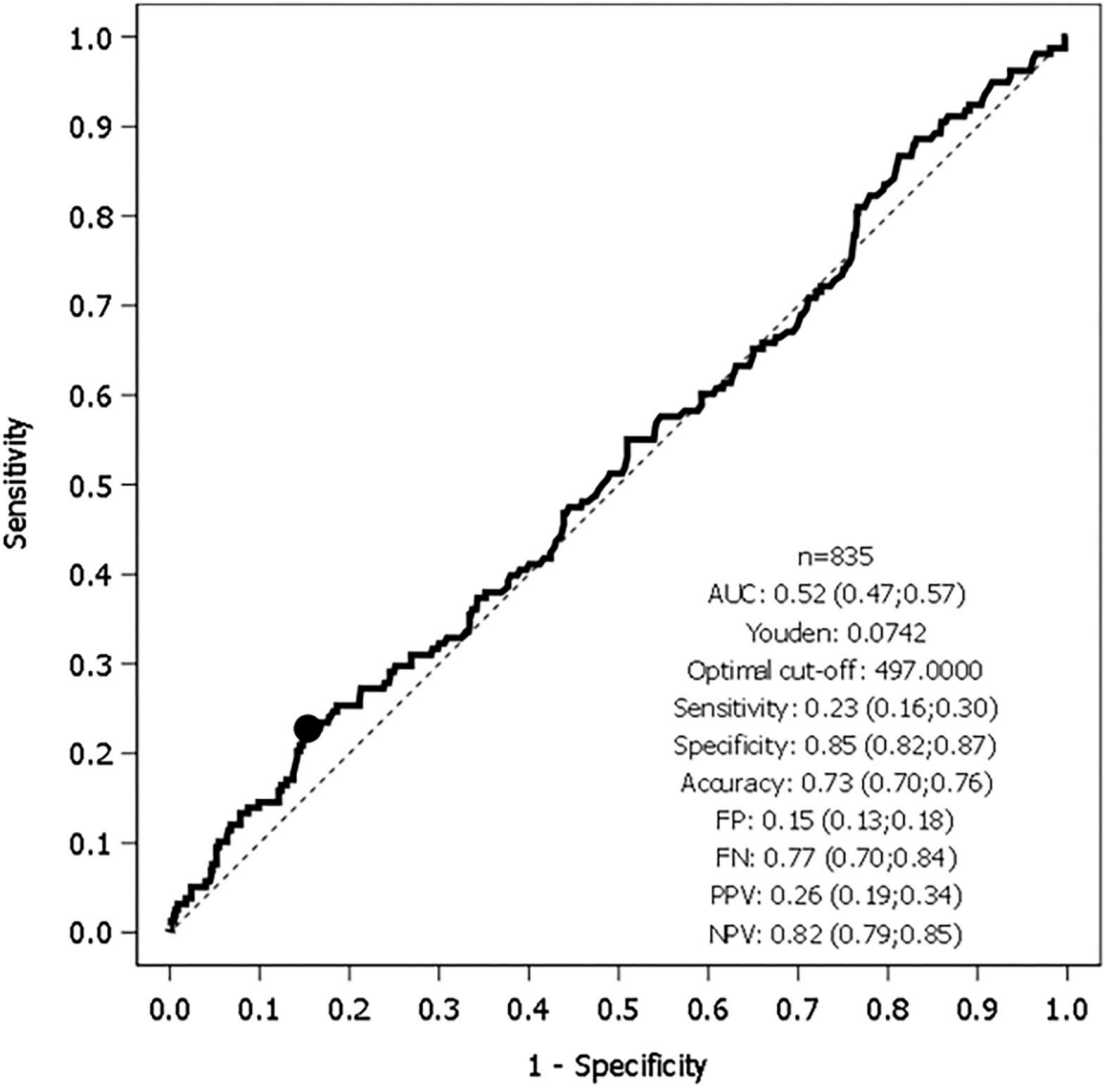
Receiver operating characteristic (ROC) curve of Krebs von de Lungen-6 circulating serum levels (sKL-6) and respiratory worsening (*n* 835).

**Figure 2:**
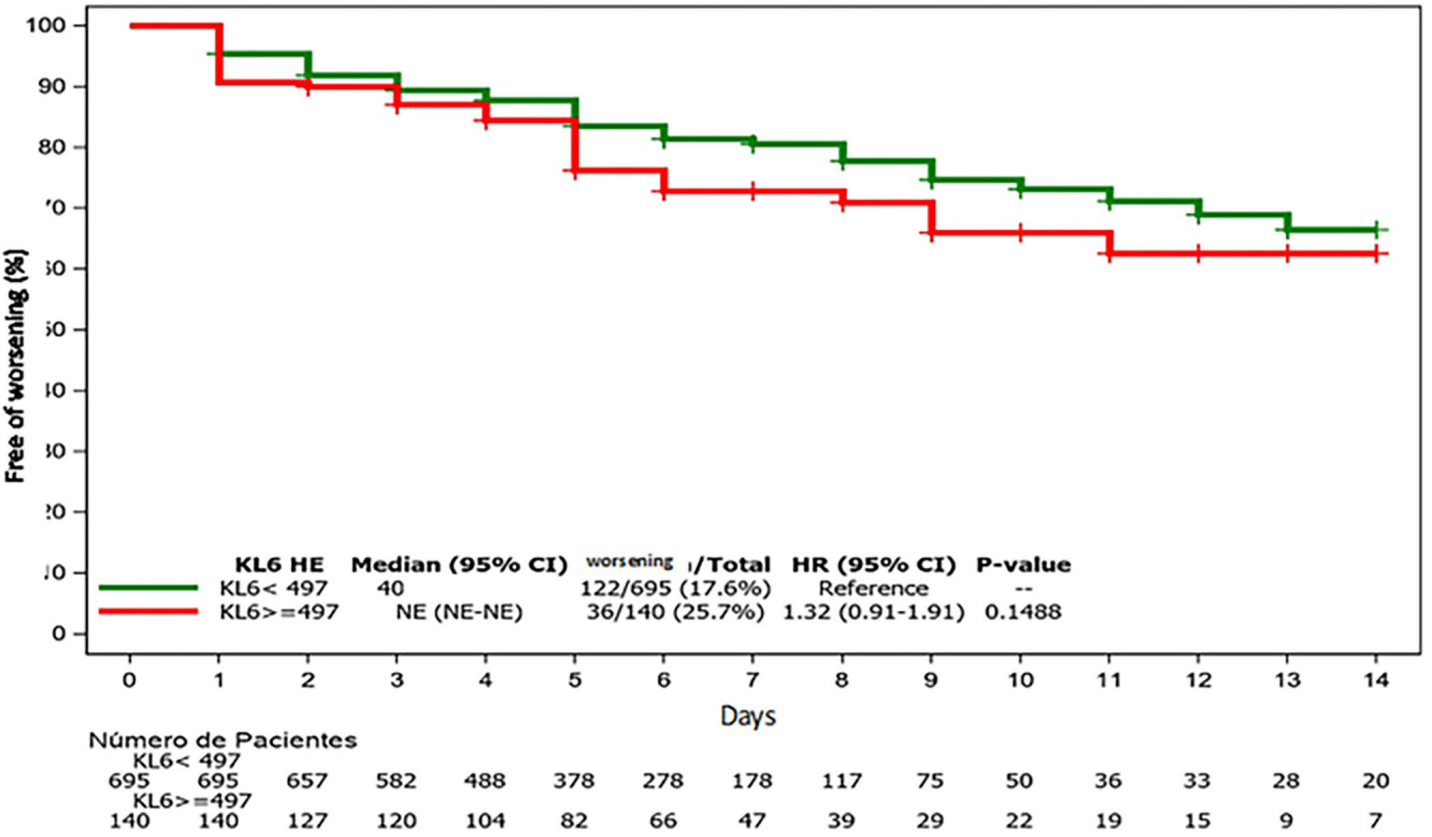
Free survival of respiratory worsening since admission (baseline sKL-6).

The best cutoff baseline sKL-6 level discriminating patients with or without impending respiratory function worsening was 497 U/ml [AUC 0.52; 23% sensitivity, 85% specificity; 26% PPV; and 82% NPV; (Figure 1)]. Patients with sKL-6≥ 497 U/ml had a 32% higher risk of respiratory function worsening since admission (*p* = 0.15; Figure 2). The worsening likelihood did not increase when symptoms onset was considered.

### Peak sKL-6 levels and respiratory function worsening

Considering that sKL-6 increased in all patients and the high number of serial samples, we calculated the best cutoff point in the peak (max) sKL-6 level to improve the AUC. The best cutoff point of the peak sKL-6 level to discriminate patients was 408 U/ml (AUC 0.56; 46% sensitivity; 65% specificity; 24% PPV; 84% NPV).

A peak sKL-6 level > 408 U/ml was not associated with a higher likelihood of respiratory function worsening. Patients with a peak sKL-6≥ 408 U/ml had a 30% higher risk of respiratory function worsening from admission (*p* = 0.098), but a trend in the likelihood of increased worsening was found when symptom onset was considered (*p* = 0.067).

### Use of sKL-6 in CTREE algorithm

**Figure 3:**
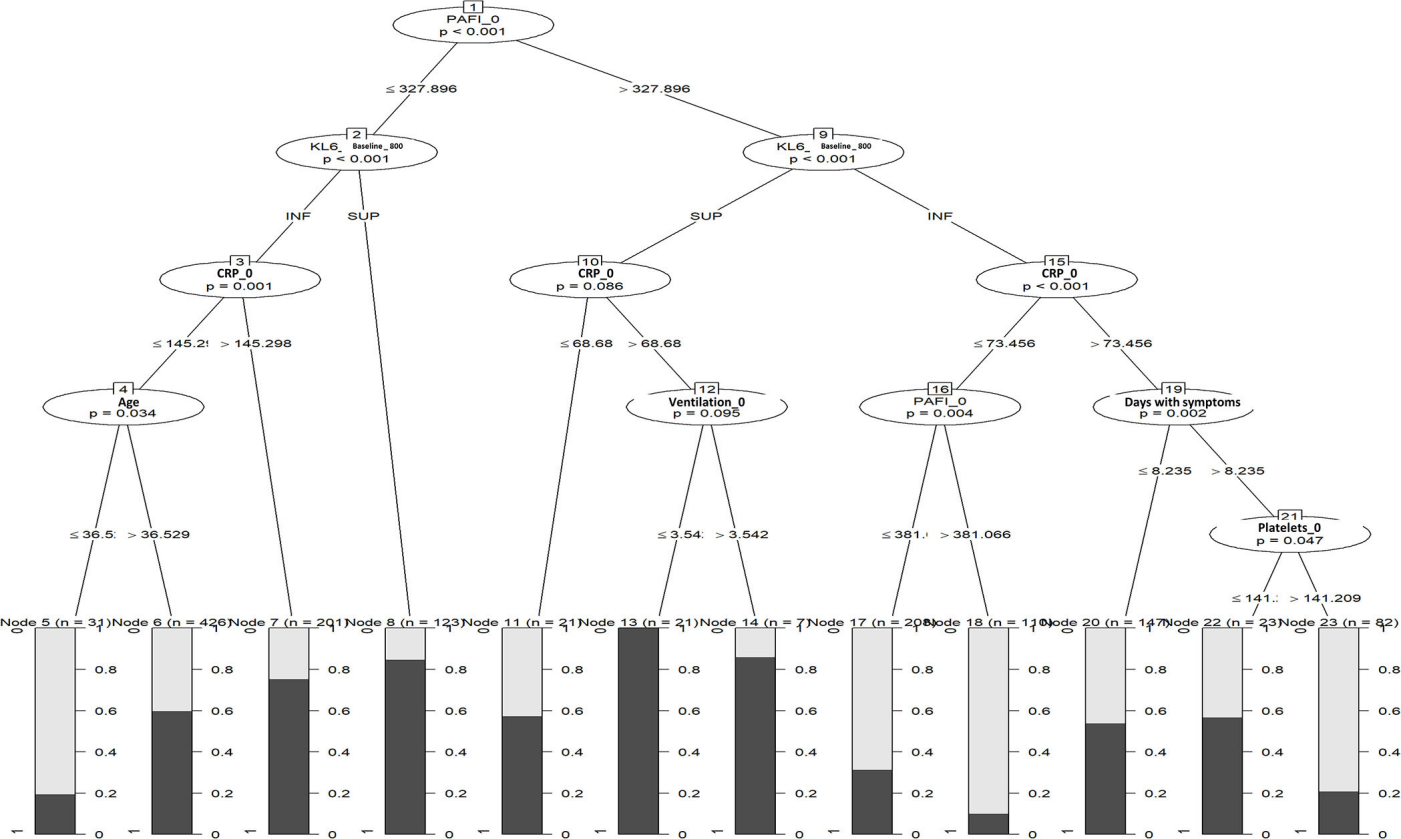
Unbiased conditional inference trees (CTREE) with baseline sKL-6.

Figure 3 shows the CTREE algorithm according to the prevalence of worsening. The most important variables associated with impending worsening were mentioned in decreasing order of importance: PAFI (*p* < 0.001), C-reactive protein (CRP) (*p* < 0.001), days from disease onset (*p* = 0.004), age (*p* = 0.005), and platelet count (*p* = 0.029). sKL-6 and peak sKL-6 level were not identified as prognostic factors for disease worsening.

## Discussion

We analyzed the value of sKL-6 circulating levels as predictors of respiratory worsening in a large population of hospitalized patients with mild COVID-19. We did not find differences between baseline sKL-6 levels in patients with or without respiratory impairment during their hospital stay, and we did not find baseline or peak sKL-6 to be a reliable biomarker of respiratory function worsening in patients with mild COVID-19. However, we did observe that the last sKL-6 values increased significantly in both groups and that this increase was higher in patients that worsened.

Considering the dramatic worldwide impact of the COVID-19 pandemic on healthcare systems, it is imperative to find a useful tool to discriminate which patients will worsen before a flare occurs. This is especially crucial in a period of the pandemic, given the pressures of resource allocation.

KL-6 and sKL-6 have recently emerged as potential biomarkers in ILDs, based on their specificity translating damage and regeneration in type II pneumocytes (33). It is well known that the viral infection of alveolar epithelial cells by SARS-CoV-2 is a crucial step in the development of the disease (34). We hypothesized that injured type II pneumocytes express excess KL-6, which spills over into systemic circulation due to the increased permeability of the alveolar-capillary membrane. A correlation between lung injury and sKL-6 levels has been demonstrated in ILDs where pneumocyte and alveolar-capillary membrane damage is observed (15–20, 35–39). Similar findings have been reported in 28 patients with ARDS who had higher median sKL-6 levels than nine ventilated controls of matched illness severity and 10 healthy donors (21).

Studies that analyze the effect of sKL-6 in patients with COVID-19 are scarce, and most have limitations in their interpretation of findings due to several factors. A recent meta-analysis of seven studies that analyzed sKL-6 levels in severe and nonsevere patients supported the potential role of sKL-6 circulating levels as predictors of severe COVID-19 (40). But those meta-analyses were based on small retrospective studies with high heterogenicity. Our prospective observational study includes more patients than all the studies analyzed in that meta-analysis together, and this factor may be critical for a correct interpretation of the findings.

In our experience, neither the best cutoff point of baseline sKL-6 nor peak sKL-6 during hospitalization shows an acceptable AUC that would allow us to consider KL-6 as a potential biomarker for predicting respiratory deterioration in patients with mild to moderate COVID-19. A few studies have looked at the sensitivity, specificity, and predictive values of sKL-6 in COVID-19. Yamaya et al. found a sensitivity of 83.3% and a specificity of 90.5 (AUC 0.89) to predict respiratory worsening with a sKL-6 cutoff point of 684 U/ml in 356 patients with sKL-6 determinations (26). Interestingly, they found a statistical association between baseline, peak, and Δ sKL-6 levels with severe illness and survival. We also found differences in peak and Δ sKL-6, but we did not find different baseline sKL-6 levels between groups, though we did not analyze survival in our cohort. The ROC curve did not allow us to identify a reliable sKL-6 threshold to predict the worsening of COVID-19. There are several differences between the Yamaya study and ours. Unlike us, Yamaya et al. analyzed data retrospectively, and their hospital did not admit patients in critical condition. This fact may have biased their survival findings and ROC results.

d'Alessandro et al. analyzed the peripheral natural killer cells and sKL-6 as potential prognostic biomarkers of COVID-19 severity in 22 patients (24). They found higher sKL-6 levels in patients with severe COVID-19 compared with patients with mild infection or a healthy group. The best cutoff point of 406.5 U/ml in sKL-6 was identified using the ROC analysis (83% sensitivity and 89% specificity) in their study. This study has some similarities with ours, notably a similar cutoff point. Nevertheless, they did not detail the timeline of patients' worsening, and baseline sKL-6 may therefore have been taken in patients who had already worsened. We collected baseline samples in nonseverely ill patients before respiratory function worsened to assess the usefulness of sKL-6 as a predictor of evolving trends in patients. Finally, another retrospective study compared different biomarkers in patients with 33 nonsevere and 21 severe COVID-19 and found higher sKL-6 levels in the severe group with a peak level 1 week after diagnosis (41). With an optimal cutoff value of 371 U/ml, they found a sensitivity and specificity of 85.7 and 96.6%, respectively (AUC 0.84) to assess severity in patients with COVID-19. In our prospective study with more patients (159 who worsened and 677 who did not), we observed opposite results (sensitivity, 46%, specificity 65%, PPV 24%, and NPV 84%; AUC 0.56), though with a similar threshold peak of sKL-6 (408 U/ml). More studies have analyzed the sensitivity and specificity of sKL-6 in COVID-19 (24, 25, 28, 42) but their main goals were different than ours since they analyzed the levels of this biomarker to predict irreversible ILD or death. We did not explore sKL-6 levels and their usefulness to predict outcomes after COVID-19 pneumonia.

Our study did not demonstrate the utility of sKL-6 in a composite algorithm to predict respiratory impairment in COVID-19. Our CTREE, which includes sKL-6 optimal values, did not find sufficient discriminatory power to predict which patients will worsen. This is the first study that includes unbiased conditional inference trees to analyze sKL-6 circulating levels to predict respiratory worsening. CTREE solves both the overfitting problem and the variable selection bias present in other recursive partitioning algorithms (32). To date, only a small study with 24 patients has explored a combination model with IL-6, sKL-6, and CRP values to detect patients with severe COVID-19 with promising results (43). Our more exact model does not support the use of sKL-6 in algorithm models.

There are a number of factors that may explain the failure of sKL-6 to predict respiratory function worsening. Different subtypes of COVID-19 pneumonia have been proposed (44), and the kind of lung damage and subsequent sKL-6 production can be different in those subtypes (23, 45). An example of this is provided by patients with COVID-19 pneumonia type H (high elastance, high right-to-left shunt, high lung weight, and high lung recruitability). sKL-6 could be useful as a potential biomarker of pulmonary worsening in comparison with type L (low elastance, low ventilation-to-perfusion ratio, low lung weight, and low lung recruitability) COVID-19 pneumonia (23). Unfortunately, we have no tools to know which pneumonia subtype is existent in our patients.

Our study has some limitations and weaknesses. First, we do not have a control group of patients with balanced risk factors, and we therefore only analyzed sKL-6 in hospitalized patients with mild COVID-19 pneumonia on admission. The absence of a control group does not allow us to determine whether sKL-6 could play a role in detecting patients before clinical pneumonia is present. During the outbreak, we had sKL-6 reactive limitations in Europe, which meant that we focused our study fieldwork on a significant sample of patients affected with mild to moderate pneumonia. Second, some patients may present a nonpreviously diagnosed ILD or have other diseases associated with abnormal sKL-6 levels not related to COVID-19 pneumonia. Another limitation was the fact that we did not study the correlation between sKL-6 levels and imaging tools (chest X-ray and computerized tomography) or other laboratory data. A major issue sometimes observed in patients with COVID-19 is that the cause of lung function worsening is related to thromboembolic phenomena (3% of patients with COVID-19 in our cohort), which may not directly affect KL-6 production and eventually spills over into circulation (46).

Finally, we were unable to analyze the survival rates of our patients. These analyses were not possible due to the low rate of deaths found in our cohort.

To summarize, our findings suggest that baseline sKL-6 levels do not predict respiratory worsening in patients with mild to moderate COVID-19 pneumonia. sKL-6 levels are not reliable enough to be used as a prognostic screening tool on admission to stratify which patients will likely need more intensive respiratory care. Future controlled trials with serial determinations of sKL-6 in patients with COVID-19 pneumonia and the effect of sKL-6 to predict lung fibrosis after hospitalization are warranted to elucidate its role as a potential biomarker for further clinical and therapeutic decisions.

## Data availability statement

The raw data supporting the conclusions of this article will be made available by the authors, without undue reservation.

## Ethics statement

The studies involving human participants were reviewed and approved by Comité etico de investigación científica (CEIC), Hospital de la Santa Creu i Sant Pau, Barcelona, Spain (IIBSP-COV-2020-35). Written informed consent for participation was not required for this study in accordance with the national legislation and the institutional requirements.

## Author contributions

Conceiving the research and manuscript drafting: IC, DC, HC, and PD. Patients recruitment: IC, SO, and DL. KL-6 handling: AMa, AB, and LM. Data analysis: IC and PD. All authors read and approved the final manuscript.

## Funding

This study was funded by a COVID-PoC BioCAT grant from the Health Department of the Catalan Government, Generalitat de Catalunya and supported by the Institut de Recerca de l'Hospital de la Santa Creu i Sant Pau, Barcelona, Spain. This study was partially supported either by the grant COV20/00070, Instituto de Salud Carlos III, Madrid, Spain.

## Conflict of interest

The authors declare that the research was conducted in the absence of any commercial or financial relationships that could be construed as a potential conflict of interest.

## Publisher's note

All claims expressed in this article are solely those of the authors and do not necessarily represent those of their affiliated organizations, or those of the publisher, the editors and the reviewers. Any product that may be evaluated in this article, or claim that may be made by its manufacturer, is not guaranteed or endorsed by the publisher.

## References

[1] Zhou P Yang XL Wang XG Hu B Zhang L Zhang W. A pneumonia outbreak associated with a new coronavirus of probable bat origin. Nature. (2020) 579:270–3. 10.1038/s41586-020-2951-z 32015507 PMC7095418

[2] Epidemiology Working Group for Ncip Epidemic Response CCfDC Prevention. The epidemiological characteristics of an outbreak of 2019 novel coronavirus diseases (COVID-19) in China. Zhonghua Liu Xing Bing Xue Za Zhi. (2020) 41:145–51. 10.3760cma.j.issn.0254-6450.2020.02.003 32064853 10.3760/cma.j.issn.0254-6450.2020.02.003

[3] Huang C Wang Y Li X Ren L Zhao J Hu Y. Clinical features of patients infected with 2019 novel coronavirus in Wuhan, China. Lancet. (2020) 395:497–506. 10.1016/S0140-6736(20)30183-5 31986264 PMC7159299

[4] Golpe R Blanco N Castro-Anon O Corredoira J Garcia-Pais MJ Perez-de-Llano LA. Factors associated to hospital admission in a care protocol in COVID-19. Arch Bronconeumol (Engl Ed). (2020) 56:676–7. 10.1016/j.arbr.2020.05.009 32620418 PMC7298471

[5] Drug treatments for covid-19: living systematic review and network meta-analysis. BMJ. (2021) 373:n967. 10.1136/bmj.n967 33849936

[6] Butt AA Yan P Shaikh OS Mayr FB Omer SB. Rate and risk factors for severe/critical disease among fully vaccinated persons with breakthrough SARS-CoV-2 infection in a high-risk national population. Clin Infect Dis. (2021) 10:ciab1023. 10.1093/cid/ciab1023 34893812 PMC8689859

[7] Estella A Canton ML Munoz L Higueras IR Recuerda Nunez M Tejero Aranguren J. Vaccinated patients admitted in ICU with severe pneumonia due to SARS-CoV-2: a multicenter pilot study. J Pers Med. (2021) 11:1086. 10.3390/jpm11111086 34834437 PMC8625038

[8] Tian W Jiang W Yao J Nicholson CJ Li RH Sigurslid HH. Predictors of mortality in hospitalized COVID-19 patients: a systematic review and meta-analysis. J Med Virol. (2020). 10.1002/jmv.26050 PMC7280666 32441789

[9] Guo W Li M Dong Y Zhou H Zhang Z Tian C. Diabetes is a risk factor for the progression and prognosis of COVID-19. Diabetes Metab Res Rev. (2020) 36:e3319. 10.1002/dmrr.3319 32233013 PMC7228407

[10] Wang D Li R Wang J Jiang Q Gao C Yang J. Correlation analysis between disease severity and clinical and biochemical characteristics of 143 cases of COVID-19 in Wuhan, China: a descriptive study. BMC Infect Dis. (2020) 20:519. 10.1186/s12879-020-05242-w 32677918 PMC7364396

[11] Bao C Tao X Cui W Yi B Pan T Young KH. SARS-CoV-2 induced thrombocytopenia as an important biomarker significantly correlated with abnormal coagulation function, increased intravascular blood clot risk and mortality in COVID-19 patients. Exp Hematol Oncol. (2020) 9:16. 10.1186/s40164-020-00172-4 32695551 PMC7366559

[12] Yao Y Cao J Wang Q Shi Q Liu K Luo Z. D-dimer as a biomarker for disease severity and mortality in COVID-19 patients: a case control study. J Intensive Care. (2020) 8:49. 10.1186/s40560-020-00466-z 32665858 PMC7348129

[13] Huang I Pranata R Lim MA Oehadian A Alisjahbana B. C-reactive protein, procalcitonin, D-dimer, and ferritin in severe coronavirus disease-2019: a meta-analysis. Ther Adv Respir Dis. (2020) 14:1753466620937175. 10.1177/1753466620937175 32615866 PMC7336828

[14] Munoz-Rodriguez JR Gomez-Romero FJ Perez-Ortiz JM Lopez-Juarez P Santiago JL Serrano-Oviedo L. Characteristics and risk factors associated with mortality in a multicenter spanish cohort of patients with COVID-19 pneumonia. Arch Bronconeumol. (2021) 57:34–41. 10.1016/j.arbres.2021.02.021 34629641 PMC7939995

[15] Kuwana M Shirai Y Takeuchi T. Elevated serum Krebs von den Lungen-6 in early disease predicts subsequent deterioration of pulmonary function in patients with systemic sclerosis and interstitial lung disease. J Rheumatol. (2016) 43:1825–31. 10.3899/jrheum.160339 27481907

[16] Kobayashi N Takezaki S Kobayashi I Iwata N Mori M Nagai K. Clinical and laboratory features of fatal rapidly progressive interstitial lung disease associated with juvenile dermatomyositis. Rheumatology (Oxford). (2015) 54:784–91. 10.1093/rheumatology/keu385 25288783

[17] Yokoyama A Kondo K Nakajima M Matsushima T Takahashi T Nishimura M. Prognostic value of circulating KL-6 in idiopathic pulmonary fibrosis. Respirology. (2006) 11:164–8. 10.1111/j.1440-1843.2006.00834.x 16548901

[18] Yokoyama A Kohno N Hamada H Sakatani M Ueda E Kondo K. Circulating KL-6 predicts the outcome of rapidly progressive idiopathic pulmonary fibrosis. Am J Respir Crit Care Med. (1998) 158:1680–4. 10.1164/ajrccm.158.5.9803115 9817725

[19] Sokai A Tanizawa K Handa T Kanatani K Kubo T Ikezoe K. Importance of serial changes in biomarkers in idiopathic pulmonary fibrosis. ERJ Open Res. (2017) 3:00019–2016. 10.1183/23120541.00019-2016 28875146 PMC5576222

[20] Hamai K Iwamoto H Ishikawa N Horimasu Y Masuda T Miyamoto S. Comparative study of circulating MMP-7, CCL18, KL-6, SP-A, and SP-D as disease markers of idiopathic pulmonary fibrosis. Dis Markers. (2016) 2016:4759040. 10.1155/2016/4759040 27293304 PMC4886062

[21] Sato H Callister ME Mumby S Quinlan GJ Welsh KI duBois RM. KL-6 levels are elevated in plasma from patients with acute respiratory distress syndrome. Eur Respir J. (2004) 23:142–5. 10.1183/09031936.03.00070303 14738246

[22] Xue M Zheng P Bian X Huang Z Huang H Zeng Y. Exploration and correlation analysis of changes in Krebs von den Lungen-6 levels in COVID-19 patients with different types in China. Biosci Trends. (2020). 10.5582/bst.2020.03197 32565512

[23] Nakamura H Miyagi K Otsuki M Higure Y Nishiyama N Kinjo T. Serum KL-6 can distinguish between different phenotypes of severe COVID-19. J Med Virol. (2020). 10.1002/jmv.26268 PMC7361808 32633842

[24] d'Alessandro M Cameli P Refini RM Bergantini L Alonzi V Lanzarone N. Serum KL-6 concentrations as a novel biomarker of severe COVID-19. J Med Virol. (2020) 92:1902–14. 10.1002/jmv.26087 32470148 PMC7283867

[25] Xue M Zhang T Chen H Zeng Y Lin R Zhen Y. Krebs Von den Lungen-6 as a predictive indicator for the risk of secondary pulmonary fibrosis and its reversibility in COVID-19 patients. Int J Biol Sci. (2021) 17:1565–73. 10.7150/ijbs.58825 33907520 PMC8071769

[26] Yamaya T Hagiwara E Baba T Kitayama T Murohashi K Higa K. Serum Krebs von den Lungen-6 levels are associated with mortality and severity in patients with coronavirus disease 2019. Respir Investig. (2021) 59:596–601. 10.1016/j.resinv.2021.04.002 33965361 PMC8075813

[27] Deng K Fan Q Yang Y Deng X He R Tan Y. Prognostic roles of KL-6 in disease severity and lung injury in COVID-19 patients: a longitudinal retrospective analysis. J Med Virol. (2021) 93:2505–12. 10.1002/jmv.26793 33433006 PMC8013517

[28] Chen H Qin R Huang Z Luo W Zheng P Huang H. Clinical relevance of serum Krebs von den Lungen-6 levels in patients with coronavirus disease 2019. Cytokine. (2021) 148:155513. 10.1016/j.cyto.2021.155513 34507246 PMC7997619

[29] Beigel JH Tomashek KM Dodd LE. Remdesivir for the treatment of Covid-19 - preliminary report. Reply N Engl J Med. (2020) 383:994. 10.1056/NEJMoa2007764 32649078

[30] Brown SM Grissom CK Moss M Rice TW Schoenfeld D Hou PC. Nonlinear imputation of Pao2/Fio2 from Spo2/Fio2 among patients with acute respiratory distress syndrome. Chest. (2016) 150:307–13. 10.1016/j.chest.2016.01.003 26836924 PMC4980543

[31] Brown SM Duggal A Hou PC Tidswell M Khan A Exline M. Nonlinear imputation of PaO2/FIO2 from SpO2/FIO2 among mechanically ventilated patients in the ICU: a prospective, observational study. Crit Care Med. (2017) 45:1317–24. 10.1097/CCM.0000000000002514 28538439 PMC5511089

[32] Strobl C Boulesteix AL Zeileis A Hothorn T. Bias in random forest variable importance measures: illustrations, sources and a solution. BMC Bioinformatics. (2007) 8:25. 10.1186/1471-2105-8-25 17254353 PMC1796903

[33] Ishikawa N Hattori N Yokoyama A Kohno N. Utility of KL-6/MUC1 in the clinical management of interstitial lung diseases. Respir Investig. (2012) 50:3–13. 10.1016/j.resinv.2012.02.001 22554854

[34] Domingo P Mur I Pomar V Corominas H Casademont J de Benito N. The four horsemen of a viral Apocalypse: the pathogenesis of SARS-CoV-2 infection (COVID-19). EBioMedicine. (2020) 58:102887. 10.1016/j.ebiom.2020.102887 32736307 PMC7387269

[35] Kohno N Awaya Y Oyama T Yamakido M Akiyama M Inoue Y. KL-6, a mucin-like glycoprotein, in bronchoalveolar lavage fluid from patients with interstitial lung disease. Am Rev Respir Dis. (1993) 148:637–42. 10.1164/ajrccm/148.3.637 8368634

[36] Elhai M Avouac J Allanore Y. Circulating lung biomarkers in idiopathic lung fibrosis and interstitial lung diseases associated with connective tissue diseases: where do we stand? Semin Arthritis Rheum. (2020) 50:480–91. 10.1016/j.semarthrit.2020.01.006 32089354

[37] Yamakawa H Hagiwara E Kitamura H Yamanaka Y Ikeda S Sekine A. Serum KL-6 and surfactant protein-D as monitoring and predictive markers of interstitial lung disease in patients with systemic sclerosis and mixed connective tissue disease. J Thorac Dis. (2017) 9:362–71. 10.21037/jtd.2017.02.48 28275485 PMC5334095

[38] Ma H Lu J Song Y Wang H Yin S. The value of serum Krebs von den lungen-6 as a diagnostic marker in connective tissue disease associated with interstitial lung disease. BMC Pulm Med. (2020) 20:6. 10.1186/s12890-019-1043-z 31915006 PMC6950990

[39] Kim HC Choi KH Jacob J Song JW. Prognostic role of blood KL-6 in rheumatoid arthritis-associated interstitial lung disease. PLoS ONE. (2020) 15:e0229997. 10.1371/journal.pone.0229997 32163457 PMC7067443

[40] Naderi N Rahimzadeh M. Krebs von den Lungen-6 (KL-6) as a clinical marker for severe COVID-19: a systematic review and meta-analyses. Virology. (2022) 566:106–13. 10.1016/j.virol.2021.11.006 34896901 PMC8642780

[41] Awano N Inomata M Kuse N Tone M Takada K Muto Y. Serum KL-6 level is a useful biomarker for evaluating the severity of coronavirus disease 2019. Respir Investig. (2020) 58:440–7. 10.1016/j.resinv.2020.07.004 32863199 PMC7441928

[42] Scotto R Pinchera B Perna F Atripaldi L Giaccone A Sequino D. Serum KL-6 could represent a reliable indicator of unfavourable outcome in patients with COVID-19 pneumonia. Int J Environ Res Public Health. (2021) 18:18:2078. 10.3390/ijerph18042078 33672761 PMC7924557

[43] Bergantini L Bargagli E. d'Alessandro M Refini RM Cameli P Galasso L. Prognostic bioindicators in severe COVID-19 patients. Cytokine. (2021) 141:155455. 10.1016/j.cyto.2021.155455 33548798 PMC7843114

[44] Gattinoni L Chiumello D Caironi P Busana M Romitti F Brazzi L. COVID-19 pneumonia: different respiratory treatments for different phenotypes? Intensive Care Med. (2020) 46:1099–102. 10.1007/s00134-020-06033-2 32291463 PMC7154064

[45] Japan EfC. Nationwide system to centralize decisions around ECMO use for severe COVID-19 pneumonia in Japan (Special Correspondence). J Intensive Care. (2020) 8:29. 10.1186/s40560-020-00445-4 32341785 PMC7180634

[46] Benito N Filella D Mateo J Fortuna AM Gutierrez-Alliende JE Hernandez N. Pulmonary thrombosis or embolism in a large cohort of hospitalized patients with Covid-19. Front Med (Lausanne). (2020) 7:557. 10.3389/fmed.2020.00557 32984388 PMC7477312

